# Embryo transfer can be performed in either uterine horn. Two successive pregnancies in a patient with uterus didelphys: a case report

**DOI:** 10.1186/s13256-021-02884-5

**Published:** 2021-06-14

**Authors:** Zakwan Khrait

**Affiliations:** Consultant, Reproductive Endocrinology and Infertility, FAKIH IVF Center, Dubai, UAE

**Keywords:** Uterus didelphys, Mullerian ducts, IVF, Embryo transfer, Case report

## Abstract

**Background:**

Uterus didelphys results from a failure in Mullerian duct fusion and may be associated with complete or partial vaginal septa. Most cases of uterus didelphys are discovered incidentally during the workup of infertility or recurrent miscarriage. The incidence of uterus didelphys has been reported to be 0.2% in the infertile population.

**Case presentation:**

A 35-year-old white Arab woman, gravida 0, parity 0, with a history of primary infertility of 8 years (a well-known male factor) presented to our infertility center. She was diagnosed as having uterus didelphys with severe male factor. The patient had three previous failed *in vitro* fertilization/intracytoplasmic sperm injection cycles outside our center.

This is a case report of an infertile woman with uterus didelphys who conceived twice following single embryo transfer in both uterine horns successively. After the first successful pregnancy in the left uterine horn, the initial decision was to transfer the embryo to the same horn (left) because of the previous successful transfer. The very deep and long vagina forced us to reach one of the cervices, in which the embryo was placed in the right uterine horn, followed by the second successful pregnancy in the other, smaller horn. The patient gave her informed consent, both verbal and written, to use her information and images for medical publication.

**Conclusion:**

Pregnancy is possible in women with uterus didelphys using single embryo transfer in *in vitro* fertilization/intracytoplasmic sperm injection cycles. It is recommended that both horns be given a chance.

## Introduction

The uterus forms during embryogenesis by fusion of the two paramesonephric ducts (Mullerian ducts). The two Mullerian ducts normally fuse to form a single uterine body. Complete fusion is completed between the 10th and 17th weeks of intrauterine development.

It is obvious that a variety of malformations may appear. Several degrees of duplication of the uterus are possible, ranging from complete duplication of the uterus, cervix, and vaginal canal (that is, uterus didelphys with a septate vagina) to the simple arcuate uterus. The prevalence of uterine anomalies is 6.7% in the general population and approximately 7.3% in the infertile population [1].

Uterus didelphys results from failed Mullerian duct fusion and may be associated with complete or partial vaginal septa. Most cases of uterus didelphys are discovered incidentally during infertility or recurrent miscarriage workup. The incidence of uterus didelphys has been reported to be 0.2% in the infertile population [2].

Though the delivery of a normal infant and, very rarely, twins is possible in a patient with uterus didelphys [3], the usual history is one of abortion or premature labor. Patients with uterus didelphys belong to a high-risk group and deserve particular prenatal care. Therefore, the clinical management of these patients is of great importance.

Due to the extreme rarity of this condition, only few reports describe how to deal with this case regarding embryo transfer in *in vitro* fertilization (IVF). Embryo transfer should be done to each uterine horn in women with didelphys anomaly, making it easy for the infertility specialist to consider a single embryo transfer.

## Case presentation

**Fig. 1:**
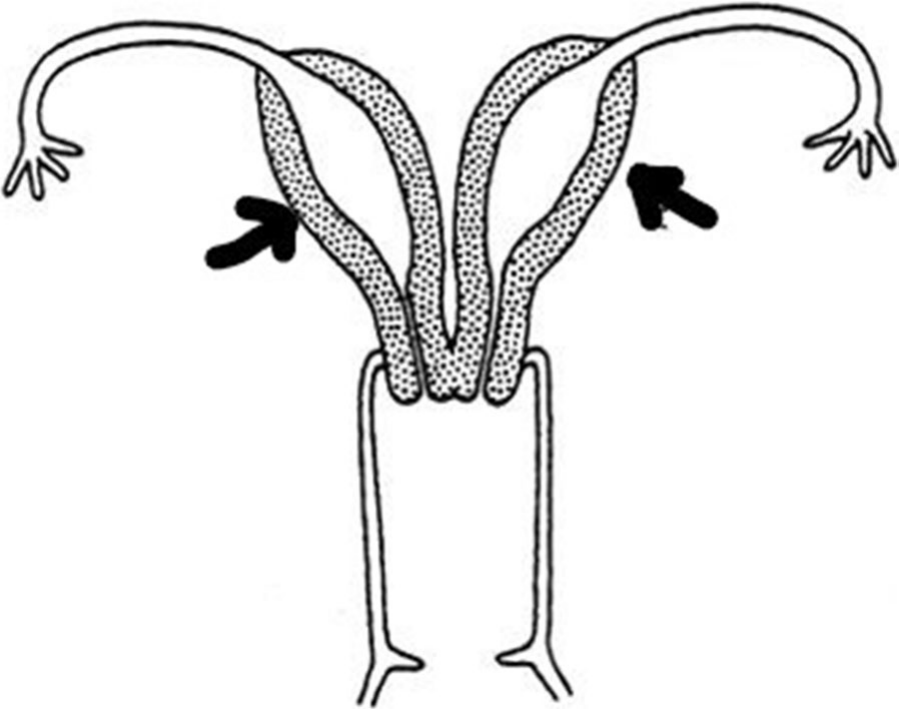
Illustration of uterus didelphys in our clinical case. The arrows are pointing to the two horns of Didelphys uterus

**Fig. 2:**
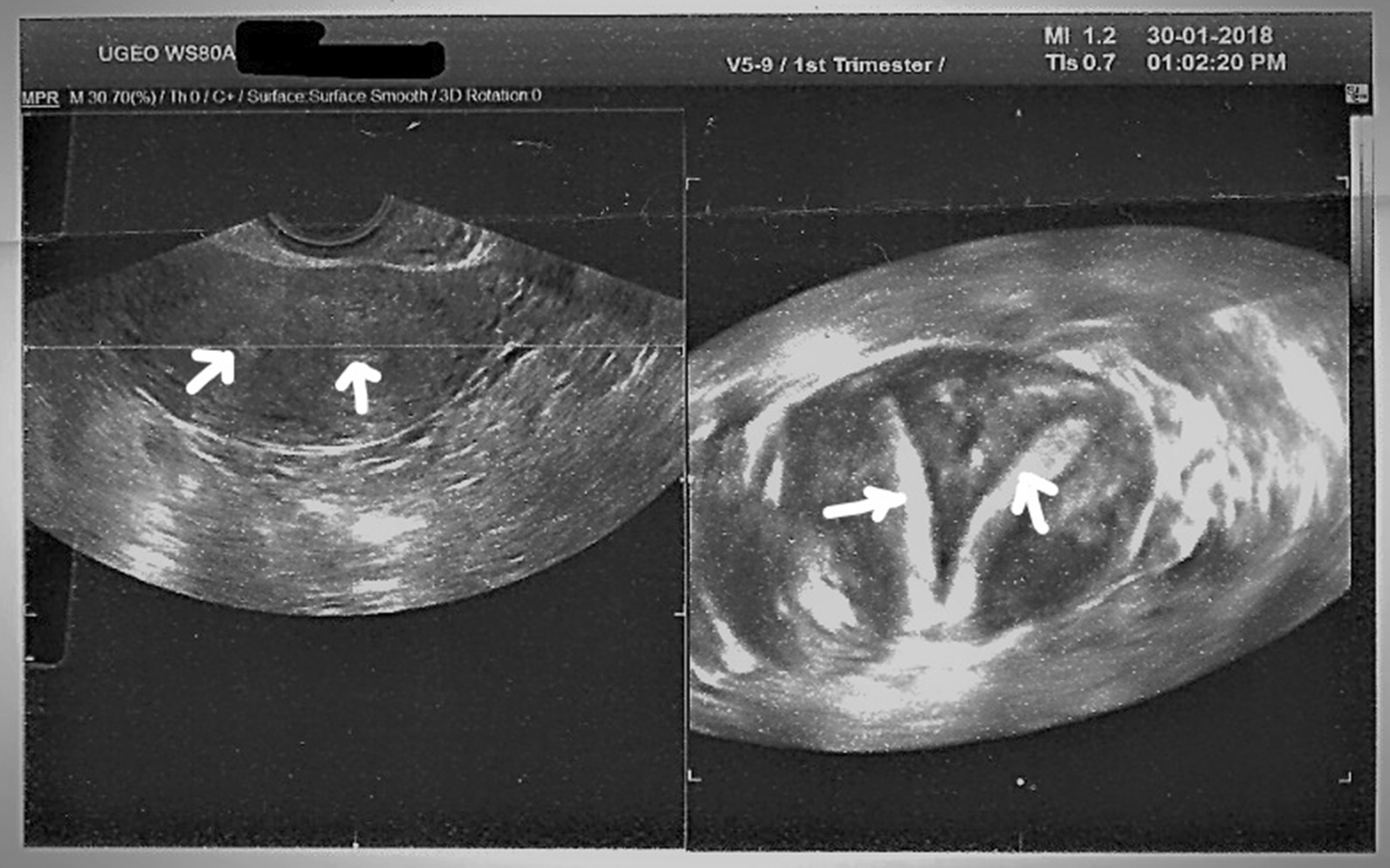
3D ultrasound of uterus didelphys in our clinical case at the time of presentation. The arrows are pointing to the two endometrial cavities of each of the two horns

A 35-year-old nulliparous Arab woman with an 8-year history of primary infertility presented to our infertility center. During these 8 years, she was diagnosed with uterus didelphys with two cervices and no vaginal septum (Fig. 1). She underwent hysterosalpingogram (HSG), three-dimensional ultrasound (3D US), and diagnostic laparoscopy, which proved the diagnosis (Fig. 2). Hence, no magnetic resonance imaging (MRI) was done since the 3D US was enough to determine the sizes of both horns, which showed minor differences in dimensions.

She had no specific history, apart from her irregular period explained by the diagnosis of polycystic ovary syndrome (PCOS), was not a smoker, and did not consume alcohol. Her weight was 87 kg and her height 158 cm.

Her spouse was 44 years old, with uncontrolled type 2 diabetes, a recent history of coronary angioplasty and stents, and chronic hypertension.

They had three previous failed intracytoplasmic sperm injection (ICSI) trials outside our center years prior due to an indication for a male factor (oligo-astheno-teratozoospermia).

The couple was very anxious, depressed, and distressed at the first consultation, to which they brought a new semen analysis showing azoospermia.

The male, who had a medical condition, declined any intervention to extract the sperm surgically per recommendations from the anesthesiologists and cardiologists. A diagnosis of retrograde ejaculation was made, as well as a plan for isolation of the sperm from a urine sample after the preparation. Three vials of frozen motile sperm were obtained before embarking on the fourth IVF trial.

Four blastocysts were obtained from the first IVF/ICSI antagonist cycle in February 2018 (Table 1), one of which (4AA) was transferred to the left uterus, and surplus freezing was done. The patient tolerated the procedure very well with no need for sedation or anesthesia. Her vital signs were normal (blood pressure 118/67 mmHg, heart rate 73 beats per minute, temperature 36.7 °C).

**Table 1:** Timeline of the first IVF cycle, 2018

	28/01/2018	06/02/2018	23/02/2018	18/03/2018
	CD2	Triggering day	Day 10 post ET	First pregnancy scan
AMH	3.08	–	–	
FSH (mIU/ml)	5.1	–	–	
LH (mIU/ml)	4.9	1.7	–	
E2 (pg/ml)	22	2060	–	
P4 (ng/ml)	>0.5	0.40	23.5	Single intrauterine gestational sac in the left horn. Fetal pole = 6±6 weeks Heart beat positive
b-hCG (mIU/ml)	>0.1	–	214	
US findings	Endometrium: thin >4.5 mm	ET = triple line 11.6 mm horn 1 (left) 11 mm horn 2 (right)	-	

Both horns had a highly suitable endometrium, with a triple line with an average thickness of 10.3 mm. The left horn was chosen because it was more accessible to the left cervix at the moment of embryo transfer. It was planned to transfer one embryo, and the couple was counseled by our high-risk-pregnancy consultant to improve the obstetrics outcome for this uterine anomaly.

The first successful IVF journey ended with no need for cerclage and with no obstetrical complication by an elective C-section at 35 weeks for a baby girl of 2800 g weight, 46 cm length, 31.5 cm head circumference, and 7/10 Apgar score at delivery.

The couple visited our infertility center again in 2020 with the goal of conceiving a second child. The first child was with them, showing very good progress, normal development, and intelligence as per her parents. They extended their request to transfer one of the frozen embryos.

The endometrium was prepared by hormone replacement therapy (HRT) protocol for embryo transfer for the frozen embryo transfer (FET) cycle using 6 mg ethinyl estradiol daily starting from second day of period. Progesterone was added on day 14, when reaching triple-line endometrium with thickness of 11.1 mm. One blastocyst was thawed and survived. The initial decision was to transfer the embryo to the same horn (left) because of the previous successful transfer. The very deep and long vagina forced us to reach one of the cervices. The outer sheet of the embryo transfer catheter was used and showed that we were targeting the right horn.

**Fig. 3:**
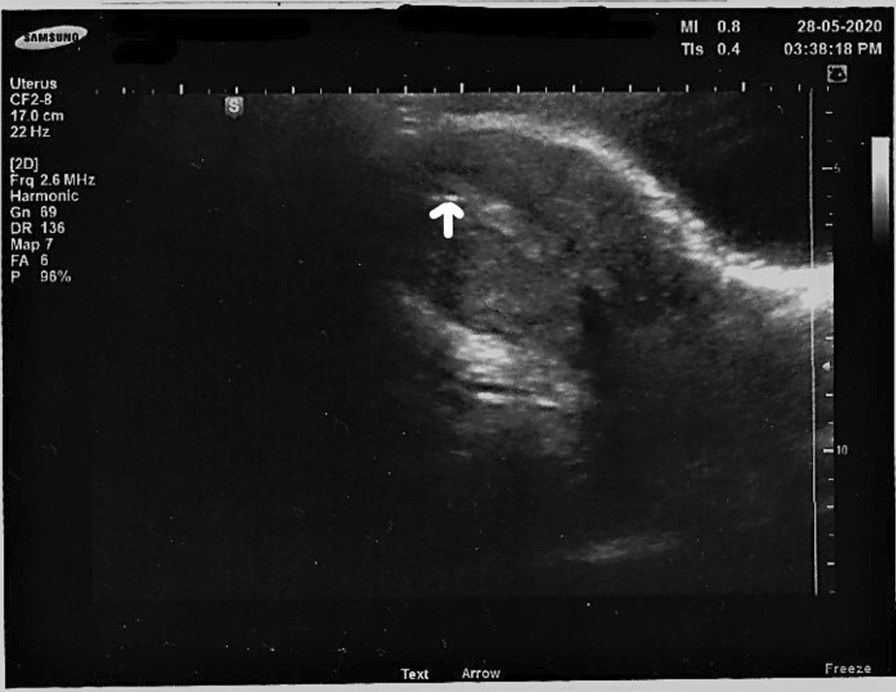
Ultrasound-guided embryo transfer. The arrow is pointing to the embryo transfer (air bubble) 1.2 cm from fundus

Embryo transfer proceeded successfully and smoothly (Fig. 3), in which the embryo was placed in the right uterus without changing or manipulating the anatomy, and the embryo was placed 1 cm from fundus. The patient tolerated the procedure well, and the couple was counseled properly.

Nine days later, her beta human chorionic gonadotropin (b-hCG) level was 258 mIU/ml. A single gestational sac with a viable pregnancy (positive heart beat) was observed in the right uterus, and the patient was referred to our high-risk-pregnancy specialist for follow-up. An elective C-section was performed at 34 weeks for a baby boy of 2650 g weight, 47 cm length, 31 cm head circumference, and 6/10 Apgar score at delivery.

## Discussion

Mullerian duct anomalies affect between 0.1% and 3% of women. In the most extreme form of Mullerian duct nonfusion, uterus didelphys results in complete duplication of the uterus, cervix, and vagina. This anomaly accounts for 11% of all uterine malformations. Heinonen estimated that one in every thousand women will have a didelphys uterus; such women are typically asymptomatic [4].

The association between having a Mullerian duct anomaly and fertility is debatable. The body of literature on didelphys uterus, although limited, generally shows that the fertility of women with untreated didelphys uterus is better than that of women with other Mullerian duct abnormalities (MDAs) but still less than that of women with normal uterine anatomy. A review by Grimbizis demonstrated that the incidence of MDAs in infertile patients (3.4%) was similar to that of the general population and/or fertile women (4.3%), which they concluded demonstrated that MDAs may not have a negative impact on fertility [5].

However, some studies demonstrate the impact of didelphys uterus on reproductive outcome [6]. A large retrospective longitudinal study of 3181 patients by Raga *et al. *demonstrated poor reproductive performance in women with didelphys uteri, with a higher rate of preterm delivery and spontaneous abortion, and the lowest chance of having a term delivery compared with the other MDAs [7].

Didelphys uterus has been shown in many case reports to allow the physician to plan a clinical approach better than in this case, especially in IVF programs. From a methodological point of view, it would have been optimal to transfer one embryo to each hemiuterus at the same time. There are reported cases of women with didelphys uteri pregnant with twins or triplets, demonstrating the ability to conceive and support the healthy growth of a fetus in either one of the uteri [8, 9].

In contrast, elective single embryo transfer is especially critical in patients with uterine pathologies since risks associated with multiple pregnancies are even more serious [10], especially after using endometrial receptivity analysis (ERA) tests in IVF treatments to analyze the receptivity of the endometrium of both hemiuteri and to determine in which to perform embryo transfer [11].

In our case, the uterine anomaly was not the cause of infertility; rather, an added factor, that is, a severe male factor (retrograde ejaculation), was the main cause of primary infertility and the reason we performed ICSI/ET. The choice of which uterine horn to transfer the embryo to was not made after hysteroscopy consideration [12] or even after ERA tests [11]. It was made owing to the difficulties in reaching the estimated cervix for the left formerly pregnant horn of the uterus.

We consider that this case report can be useful for the scientific community, clinicians, and patients in a similar situation. Embryo transfer should be done to each uterine horn in women with didelphys anomaly, making it easy for the infertility specialist to consider a single embryo transfer.

## Conclusion

Due to the extreme rarity of this condition, only few reports have described how to deal with this case with regard to embryo transfer in IVF. We determined what can help both the patient and the IVF specialist to deal with such cases.

Pregnancy is possible in women with uterus didelphys using single embryo transfer in IVF/ICSI cycles. It is recommended that both horns be given a chance, especially in difficult cases in which the estimated cervix is reached during embryo transfer. The initial detailed ultrasound is considered beneficial in such women to choose which horn is a better choice for embryo transfer and to exclude any other abnormalities that might interfere with implantation. Hence, an embryo can be transplanted into either uterine horn.

## Data Availability

Yes.

## Abbreviations

IVF: *In vitro* fertilization
ICSI: Intracytoplasmic sperm injection
3D US: Three-dimensional ultrasound
ET: Embryo transfer
FET: Frozen embryo transfer
HRT: Hormone replacement therapy
MDAs: Mullerian duct anomaly
